# Case Report: Histological and Histomorphometrical Results of a 3-D Printed Biphasic Calcium Phosphate Ceramic 7 Years After Insertion in a Human Maxillary Alveolar Ridge

**DOI:** 10.3389/fbioe.2021.614325

**Published:** 2021-04-15

**Authors:** Carlo Mangano, Alessandra Giuliani, Ilaria De Tullio, Mario Raspanti, Adriano Piattelli, Giovanna Iezzi

**Affiliations:** ^1^Independent Researcher, Gravedona, Italy; ^2^Department of Clinical Sciences, Polytechnic University of Marche, Ancona, Italy; ^3^Department of Medical, Oral and Biotechnological Sciences, University “G. D'Annunzio” of Chieti-Pescara, Chieti, Italy; ^4^Department of Medicine and Surgery, University of Insubria, Varese, Italy; ^5^Chair of Biomaterials Engineering, Catholic University of San Antonio de Murcia (UCAM), Murcia, Spain; ^6^Fondazione Villaserena per la Ricerca, Città Sant'Angelo, Italy

**Keywords:** bone substitutes, biomaterials, bone regeneration, bone augmentation, histological analysis, micro-ct, clinical research, clinical study

## Abstract

**Introduction:** Dental implant placement can be challenging when insufficient bone volume is present and bone augmentation procedures are indicated. The purpose was to assess clinically and histologically a specimen of 30%HA-60%β-TCP BCP 3D-printed scaffold, after 7-years.

**Case Description:** The patient underwent bone regeneration of maxillary buccal plate with 3D-printed biphasic-HA block in 2013. After 7-years, a specimen of the regenerated bone was harvested and processed to perform microCT and histomorphometrical analyses.

**Results:** The microarchitecture study performed by microCT in the test-biopsy showed that biomaterial volume decreased more than 23% and that newly-formed bone volume represented more than 57% of the overall mineralized tissue. Comparing with unloaded controls or peri-dental bone, Test-sample appeared much more mineralized and bulky. Histological evaluation showed complete integration of the scaffold and signs of particles degradation. The percentage of bone, biomaterials and soft tissues was, respectively, 59.2, 25.6, and 15.2%. Under polarized light microscopy, the biomaterial was surrounded by lamellar bone. These results indicate that, while unloaded jaws mimicked the typical osteoporotic microarchitecture after 1-year without loading, the BCP helped to preserve a correct microarchitecture after 7-years.

**Conclusions:** BCP 3D-printed scaffolds represent a suitable solution for bone regeneration: they can lead to straightforward and less time-consuming surgery, and to bone preservation.

## Introduction

Dental implant placement can be challenging when an insufficient bone volume is present at the recipient site (Araújo and Lindhe, 2005). Autogenous bone has been described as the gold standard in bone regeneration techniques but, due to its limitations (limited intraoral sources, tendency to rapid and partial resorption and additional surgery with increased morbidity; Yamamichi et al., 2008; Scarano et al., 2011; Iezzi et al., 2012), allografts and xenografts have been developed and proposed as suitable alternatives: they are theoretically available in limitless amounts and in different dimensions and profiles, and can be customized or combined with growth factors, hormones, drugs, and stem cells (Piattelli et al., 1996a; Pettinicchio et al., 2012; Mangano et al., 2015a; Paré et al., 2020).

Different bone substitute materials have been tested but it remains still unknown which graft material could be considered the best (Mazor et al., 2009; Iezzi et al., 2012; Pettinicchio et al., 2012; Danesh-Sani et al., 2016). Biphasic calcium phosphate ceramics (BCPs) have been reported to have a high biocompatibility and a capability to enhance cell viability and proliferation (Castilho et al., 2014; Asa'ad et al., 2016; Zeng et al., 2020). With the improvement of computer-aided design/computer-aided manufacturing (CAD/CAM) technologies it has been feasible to analyze the bone deficiency of a patient on a 3D-CT scan and to create bone grafts that fit perfectly into the receiving site (Mangano et al., 2015b; Luongo et al., 2016; Raymond et al., 2018). Several techniques have been used to produce three dimensional scaffolds [e.g., inkjet printing, stereo lithography, fused deposition modeling, and selective laser sintering (Bose et al., 2013; Hwang et al., 2017; Liu et al., 2019; Chung et al., 2020)]. These techniques allow the creation of solid constructs with an excellent pore interconnectivity, high biocompatibility, capabilities of maintaining space and, for bone regeneration procedures, they seem to be able to provide greater osteoconductivity (Carrel et al., 2016; Hwang et al., 2017; Raymond et al., 2018; Kim et al., 2020).

The purpose of the present study was to assess clinically, histologically and under high resolution X-ray tomography a specimen of 30% hydroxyapatite (HA) and 70% tricalcium phosphate (TCP) BCP-3D-printed scaffold, harvested after 7 years of healing.

### Case Description

The Ethical Committee of the Hospital of Varese, Italy approved the study protocol (N°826 del 03/10/2013). In 2013, the patient requested fixed prosthetic rehabilitation due to the lack of the second premolar and first molar of the right upper jaw. As there was lack of bone support, and the patient refused a sinus lift, it was decided to insert a dental implant in zone 1.5 with simultaneous bone regeneration of the atrophic buccal wall. The patient, who signed a written informed consent form, had undergone implant therapy with bone regeneration of the maxilla buccal plate to replace the second premolar, in 2013. Horizontal bone augmentation procedure was performed using 3D-printed biphasic HA-blocks, which were placed on the bone wall and stabilized by sutures. In CBCT 1, it is possible to see the HA 3D printed graft characterized by its particular predefined porous structure. In X-ray and CBCT 2, 4 months after regeneration, the prosthetic rehabilitation was performed with a bridge from 1.4 to 1.7. After 7 years, during which the patient had no clinical control, the patient came back with serious periodontal problems affecting the first upper right premolar (1.4) and the second upper right molar (1.7), as shown in X-ray and CBCT 3. Therefore, the patient underwent another implant surgery, to replace the first premolar in the regenerated region, and a core of regenerated bone was obtained with a trephine.

## Materials and Methods

### Scaffold Fabrication

In this study, the ceramic scaffold was made-up by the direct rapid prototyping technique dispense-plotting (Deisinger et al., 2008). The biomaterials was produced by Biomed Center (Bayrouth, Germany) following the systematic approach to the biological evaluation of the medical device, as part of the risk management process present in the ISO standard ISO 10993-1:2018 and according with ISO 14971 and ISO 13175-3: “Implants for surgery—Calcium phosphates—Part 3: Hydroxyapatite and beta-tricalcium,” as shown in the flowchart. 3D printed Biphasic HA chemical composition is manufactured under highly controlled process. A computer-generated scaffold model was designed with a cylinder-shaped outer geometry by using a 3D-CAD software. In the later sintering process, the size of the scaffold prototype was customized to the shrinkage of the ceramic material. Physical rods consisting of paste-like aqueous ceramic slurry were extruded out of a container through a jet and deposited using an industrial robot (GLT, Pforzheim, Germany), to build up the green bodies. In this study HA and TCP powders (Merck, Germany) were combined to get a biphasic powder blend with a HA/TCP weight proportion of 30/70. Thermal treatment of the raw HA powder at 900°C for 1 h and the addition of a compatible binder/dispersant system of organic additives, of 10.5 wt% relative to the amount of ceramic powder, provided to the aqueous biphasic ceramic slurry its specific rheological behavior. The rod deposition was well-ordered in x, y, and z direction to build 3D scaffolds layer by layer on a deposition platform. The rotation of the direction of the rod deposition by 60° from layer to layer produced a 3D network with an interconnecting pore arrangement. The assemblies built of ceramic slurry were dehydrated at room temperature and then sintered at 1,250°C for 1 h. The double packaging and labeling process was carried out in clean rooms (classified as ISO 6). The sterilization of the product was performed by gamma irradiation. Identification and traceability of the devices was also guaranteed.

### High-Resolution Tomography

MicroCT experiments were performed in two sessions: (1) at a laboratory-based microCT device (CISMIN Center, Polytechnic University of Marche, Ancona, Italy), achieving morphometric information on microarchitecture of the overall mineralized bone, of the newly formed bone and of the residual biomaterial, (2) at the SYRMEP microCT beamline of the ELETTRA Synchrotron Radiation Facility (Basovizza, Trieste, Italy), achieving quantitative information on osteocyte lacunae size and distribution in the newly formed bone. For laboratory-based microCT, a Skyscan 1174 (SkyScan-Bruker, Antwerp, Belgium) tomographic acquisition was set with the following parameters: voltage: 50 kV; current: 800 μA; pixel size: 6.5 μm; rotation step over 180°:0.1°; exposure time per projection: 0.1°; filter: 1 mm of Al. The absorption projection images (8 bit-TIFF) were reassembled using the NRecon software (SkyScan-Bruker, Antwerp, Belgium) to obtain a set of cross-sectional slices (8 bit-BMP), with ring artifact and beam hardening corrections. For the synchrotron-based microCT acquisition, the scans were performed using the following parameters: energy: white beam with peak energy at ~19 keV; voxel size: (890 nm^3^); rotation step over 180°:0.1°; specimen-detector distance: ~100 mm. Due to the coherence of the synchrotron source, the recorded radiographs included phase contrast signals. The method was based on the discrimination between the absorption index β and the refractive index decrement δ of the index of refraction *n* = 1 - δ + iβ in the tissues of the biopsy. The reconstruction was performed using Paganin's method (Paganin et al., 2002), together with the usual filtered back projection (FBP) algorithm. In the Paganin's method, the phase was retrieved by assuming a linear correlation between β and δ. The δ/β ratio, in the present experimental protocol, was set to 5.

The commercial software VG Studio MAX 1.2 (Volume Graphics, Heidelberg, Germany) was used to create 3D images and visualize the 3D phase distribution. X-ray contrast variations within samples turned into different peaks in the gray level scale, conforming to the several phases. The volume of each phase was acquired by multiplying the volume of a voxel by the quantity of voxels underlying the peak associated with the relevant phase. The Mixture Modeling algorithm (NIH ImageJ Plugin) was employed to threshold the histograms. Thresholded slices were used to automatically detach the new bone phase from the scaffold phase. The analyzed subvolumes were 3D portions completely embraced in the sample bulk.

The microarchitecture investigation was centered on the Parfitt structural indices (Parfitt et al., 1987): the following morphometric parameters were evaluated for the entire mineralized tissue: specific specimen volume (SV/TV—expressed as a percentage), specific specimen surface (SS/SV—per millimeter), Strut thickness (STh—expressed in micrometers), Strut number (SNr—per millimeter), and Strut spacing (SSp—expressed in micrometers). Varying bone orientation with dependency on mechanical loading, information on the eventual presence of preferential orientation(s) were extracted (Harrigan and Mann, 1984) by calculation of the anisotropy degree index (Tb.DA). Tb.DA was investigated by BoneJ Plugin (Doube et al., 2010) of the ImageJ software (Abramoff et al., 2004; Schneider et al., 2012; Rasband, 2019): it varies between 0 (perfect isotropy) and 1 (strong anisotropy). Finally, trabecular connectivity density (Tb.Conn.D) was calculated: it supplies an overall quantitative evaluation, with greater values for better-connected organizations and lower values for poorly-connected ones. For the calculation of the regenerated bone, the same quantitative descriptors, previously related to the full mineralized tissue were applied in order to quantify: overall Bone Volume (BV—mm^3^), overall Bone Surface (BS—mm^2^), Bone Volume to Total Volume ratio (BV/TV—expressed as a percentage), Bone Surface to Bone Volume ratio (BS/BV—per millimeter), Bone thickness (BTh—expressed in micrometers), Bone number (BNr—per millimeter), and Bone spacing (BSp—expressed in micrometers). The kinetics of the scaffold dissolution was also examined using again the same quantitative descriptors used to the entire mineralized tissue and to the regenerated bone [i.e.,: overall Scaffold Volume (ScV—mm^3^), overall Scaffold Surface (ScS—mm^2^), Scaffold Volume to Total Volume ratio (ScV/TV—expressed as a percentage), Scaffold Surface to Scaffold Volume ratio (ScS/ScV—per millimeter), Scaffold thickness (ScTh—expressed in micrometers), Scaffold number (ScNr—per millimeter), and Scaffold spacing (ScSp—expressed in micrometers)].

Synchrotron-based imaging allowed to achieve information on morphometric properties of the osteocyte lacunar network, with data on the mean lacunar thickness (Lc.Th), the mean lacunar volume (Lc.V), and the lacunar density (amount of lacunae per whole volume—Lc.Nr/TV).

### Histology

The biopsy was fixed in 10% buffered formalin and processed (Precise 1 Automated System; Assing, Rome, Italy) to obtain thin ground sections. The specimen was dehydrated in an ascending sequence of alcohol solutions and embedded in an ascending sequence of glycol-methacrylate resin (Technovit 7200 VLC; Kulzer, Wehrheim, Germany). After polymerization, the specimen was segmented, along its longitudinal axis, with a high precision diamond disk at about 150 μm and ground down to about 30 μm with a specifically designed grinding machine. Each slice was stained with acid fuchsin and toluidine blue and analyzed under a light microscope (Laborlux S, Leitz, Wetzlar, Germany) associated to a high-resolution video camera (3CCD, JVCKY-F55B, JVC, Yokohama, Japan) and interfaced with a monitor and PC (Intel Pentium III 1200 MMX, Intel, Santa Clara, CA, USA). This optical system was connected with a digitizing pad (Matrix Vision GmbH, Oppenweiler, Germany) and a histomorphometry software set with image capturing means (Image-Pro Plus 4.5, Media Cybernetics Inc., Immagini & Computer Snc, Milano, Italy). One single well-trained examiner (GI), who was not involved in the surgical treatment, assessed the histological results. The following outcome measures were carried out: percentages of newly formed bone, marrow spaces and residual graft particles. Birefringence was measured as a sign of transverse collagen orientation using polarized light. Collagen fibers were observed by placing the thin bone sections under an Axiolab light microscope (Laborlux S, Leitz, Wetzlar, Germany) equipped with two linear polarizers and two quarter wave plates set to have a transferred circularly polarized light. Collagen fibers aligned perfectly transverse to the course of the light spread (parallel to the specimen slice plane) appeared bright due to a modification in the refraction of existing light, while the collagen fibers aligned along the axis of light spread (perpendicular to the specimen slice plane) looked dark because no refraction happened.

## Results

### Scaffold Characterization

The sintered dispense-plotted assemblies had a typical mesh like organization with rod diameters of 300 ± 30 μm and pore sizes between the rods of about 370 ± 25 μm. By determining the geometrical density of the sintered scaffolds, a total porosity of about 60% was estimated. Relative bulk density of the sintered specimens was assessed to 99% th.d. by pycnometry. Two main material phases of the sintered ceramic were identified by semi-quantitative XRD measurements: 30% HA, 60% β-TCP, plus a small peak of α-TCP (70% of TCP in total).

### High-Resolution X-Ray Tomography

**Figure 1:**
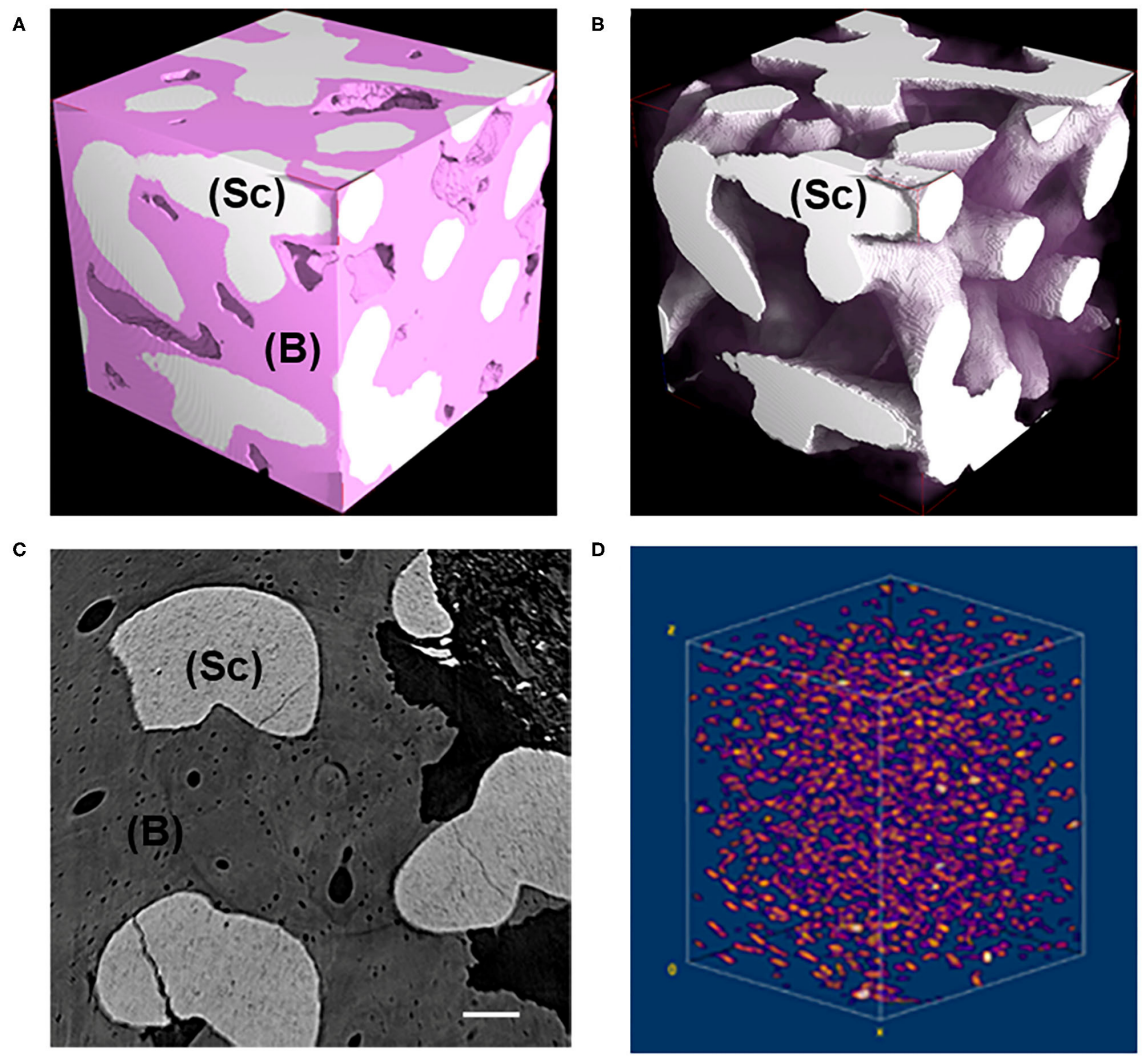
**(A,B)** Representative 3D subvolumes as obtained at desktop microCT: **(A)** block-based TCP/HA scaffold as retrieved from *in vivo* test after 7 years; **(B)** same sample of **(A)** showing the residual scaffold not fully resorbed after 7 years *in-vivo*. Bone was virtually made transparent. Pink phase: bone; White phase: residual scaffold; **(C)** Representative transversal 2D section as obtained at synchrotron microCT; light gray phase: residual scaffold; dark gray phase: bone; black phase: medullar spaces; Scale bar = 100 μm. **(D)** Representative 3D distribution of the osteocyte lacunae in a 3D bone subvolume, as obtained at synchrotron microCT. Sc, scaffold; B, bone.

MicroCT images of representative subvolumes of the Test-sample (i.e., of the maxilla biopsy grafted with the BCP and retrieved after 7 years, were shown in Figure 1). All tissues, but mineralized bone and residual scaffold, have been made virtually transparent in Figure 1A, while in Figure 1B the same subvolume was shown with also the newly formed bone made transparent for a better visualization of the residual scaffold, not fully resorbed after 7 years *in-vivo*. A transversal section and the 3D distribution of the osteocyte lacunae in a representative subvolume were respectively reported in Figures 1C,D. Numerous subvolumes, collected in different areas and completely included in the biopsy, were chosen, producing the microarchitecture data described in Tables 1, 2A.

**Table 1:** Study of microarchitecture in the test-maxillary biopsy (Test) retrieved after 7 years *in-vivo*: whole Mineralized Structure (S), Newly Formed Bone (B), and Scaffold Residuals (Sc) were considered.

	**Test**			**Ctr-Sc**
	**Whole mineralized** **structure (S)**	**Newly formed** **bone (B)**	**Scaffold residuals** **(Sc)**	**BCP** **scaffold**
Specific surface [mm^−1^]	6	19	17	13
Specific volume [%]	93.3	53.6	39.8	51.9
Mean thickness [μm]	318	107	116	147
Mean number [mm^−1^]	3	5	3	3
Mean spacing [μm]	23	93	176	146

*Comparisons are made with the BCP scaffold before in-vivo tests (Ctr-Sc)*.

**TABLE 2A:** Study of microarchitecture in the test-maxillary biopsy (Test) retrieved after 7 years *in-vivo*: comparison with peri-dental bone microarchitecture (Pd-Ctr) and with unloaded bone control (UnL-Ctr).

	**Test**	**Peri-dental^†^**	**Unloaded site^†^**
		**Pd-Ctr**	**UnL-Ctr**
SS/BV [mm^−1^]	6.0	12.0 (2.1)	21.0 (0.1)
SV/TV [%]	93.3	57.8 (0.5)	42.2 (5.4)
S.Th [μm]	318	166 (40)	104 (16)
S.Nr [mm^−1^]	3.0	3.3 (0.7)	4.0 (0.0)
S.Sp [μm]	23	120 (22)	141 (10)
Tb.DA	0.553	0.590 (0.048)	0.752 (0.010)
Tb.Conn.D [mm^−3^]	8	56 (10)	60 (56)

*Pd-Ctr and UnL-Ctr data were extracted (Iezzi et al., 2020). Mean (std.dev.) are indicated*.

† *Iezzi et al. (2012)*.

The study of the microarchitecture in the test-maxillary biopsy (Test-sample retrieved after 7 years *in-vivo*) was detailed in Table 1: the full mineralized structure (S), the newly-formed bone (B), and the scaffold residuals (Sc) were considered. A comparison was made with the BCP scaffold as produced [i.e., before the *in-vivo* test (Ctr-Sc)]. After 7 years, against a comparable number of struts, an increase in the specific volume of almost 80% was observed and the average thickness of the struts by more than 100%, together with a decrease in the specific surface of almost 54% and average spacing between struts of over 80%. Furthermore, after 7 years *in vivo*, a reduction of the biomaterial volume of more than 23% was observed and the newly formed bone volume was more than 57% of the overall mineralized volume. In this context, it has been widely accepted in literature that jawbones, 6 months after tooth extraction, were perfectly healed in healthy patients (Guralnick, 1968; Jahangiri et al., 1998). Basing on the previously shown data, also the Test-sample had to be considered healed. However, this Test-sample did not participate in mastication for 7 years; thus, it was particularly interesting to study possible alterations with the physiological conditions of the peri-dental bone (Pd-Ctr) and with unloaded controls (UnL-Ctr), (i.e., with bone biopsies spontaneously healed in 12 months after tooth extraction but not participating in mastication). This comparative study, shown in Tables 2A,B, was supported by the data of a recent study (Iezzi et al., 2020). Table 2A showed such comparison in terms of microarchitecture quantitative study: it was observed that the Test-sample turned out to be much more mineralized and bulky not only compared to UnL-Ctr, with an increase of the mineralized volume of 121%, but also compared to the peri-dental physiological context (Pd-Ctr), with an increase by over 61%. Interestingly, the anisotropy degree (DA) of the Test-sample resembled that of the peri-dental site and it was shown to be much less oriented than the UnL-Ctr samples. The increasing in terms of mineralized volume of the BCP-based Test-sample was correlated to the study of bone architecture at the length-scale pertaining the observation of the osteocyte lacunar network; the same subvolumes investigated for producing the microarchitecture data were also studied for the osteocyte lacunae 3D morphometric analysis (Table 2B): considering the standard deviations, comparable values in Test and in Control sites (both Pd-Ctr and UnL-Ctr), when evaluating Lac.V, Lac.Th, and Lac.Nr. However, the observation of the pure mean values, revealed the same values in Test-sample and the UnL-Ctr samples in terms of lacunar density (Lac.Nr), but an increased mean Lac.Nr in the physiological context of the peri-dental site (Pd-Ctr), in agreement with previous observations (Iezzi et al., 2020).

**TABLE 2B:** Three-dimensional morphometric investigation of the osteocyte lacunar network in the test-maxilla (Test) retrieved after 7 years *in-vivo*: comparison with peri-dental bone (Pd-Ctr) and with unloaded bone (UnL-Ctr).

	**Test**	**Peri-dental^†^**	**Unloaded^†^**
		**Pd-Ctr**	**Ctr**
Lac.Th [μm]	5.9 ± 0.6	5.2 ± 1.3	5.6 ± 0.7
Lac.V [μm^3^]	637 ± 152	409 ± 180	371 ± 133
Lac.Nr [× 10^3^ mm^−3^]	25.0 ± 0.5	31.4 ± 10.6	25.6 ± 6.9

*Pd-Ctr and UnL-Ctr data were extracted (Iezzi et al., 2020). Mean ± std.dev. are indicated*.

† *Iezzi et al. (2020)*.

### Histological Results

**Figure 2:**
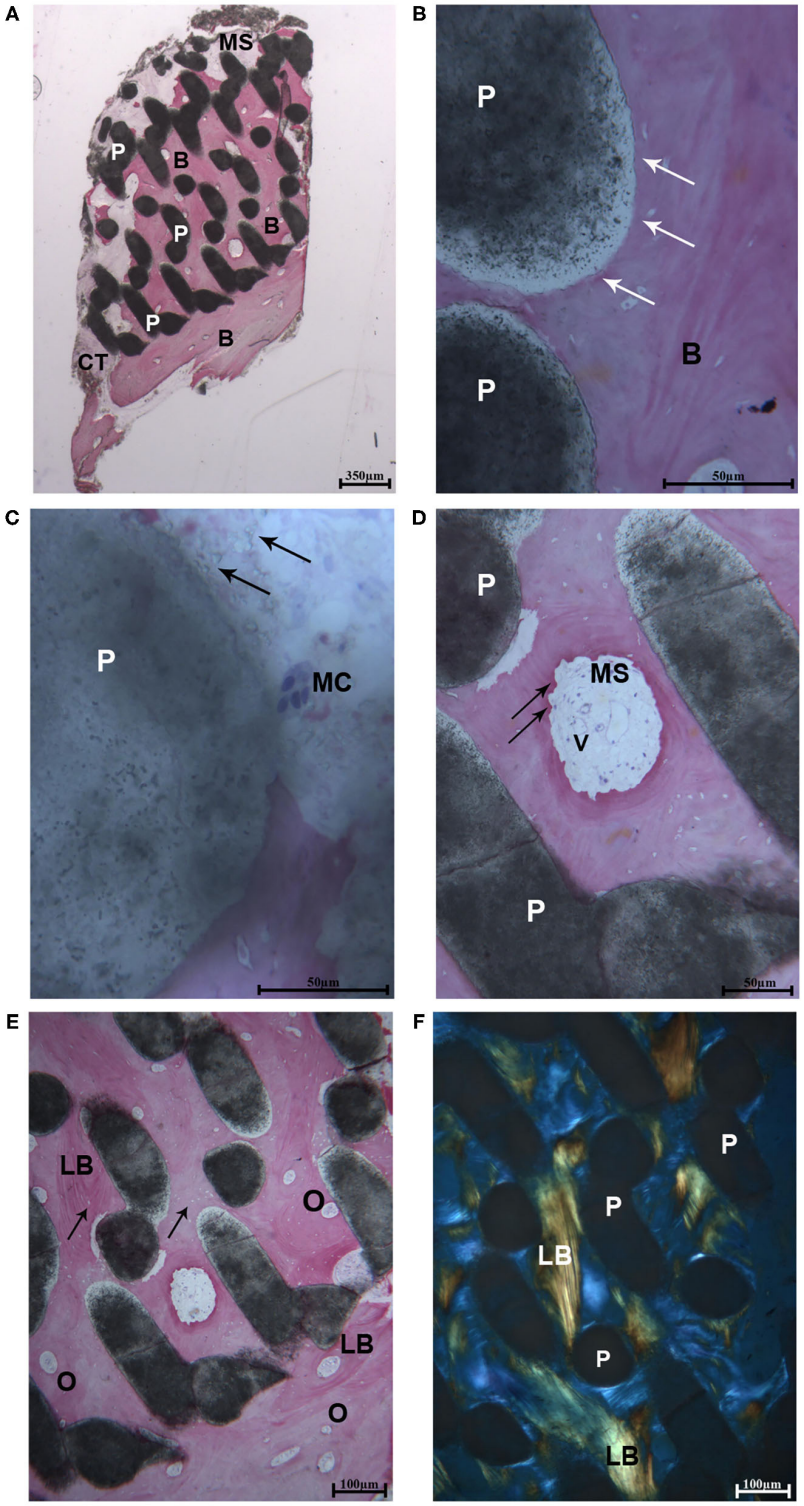
**(A)** Light microscopic ground sections of the specimen showed the residual biomaterial block (P) surrounded by mature bone **(B)**. The bone marrow (MB) and a small portion of connective tissue (CT) were present. At the bottom of the samples (occlusal region), mature bone with very small marrow spaces was shown **(B)** (Acid fuchsin-Toluidine blue 12X). **(B)** At higher-power magnification, the biomaterial particles (P) were in tight contact with the mature bone **(B)**. At the bone-biomaterial particles interface, the particles showed a lower density (black arrows) compared to their central portion (Acid fuchsin-Toluidine blue 40X). **(C)** Close to the residual biomaterial, which revealed signs of resorption (black arrows), a multinucleated giant cell was observed (MC). **(D)** In the small marrow spaces (MS), some blood vessels (V), and signs of bone remodeling were present (black arrows) (Acid fuchsin-Toluidine blue 200 and 100X). **(E)** Mature lamellar bone (LB) with small osteocyte lacunae were observed (black arrows) and many secondary osteons were detected (O). **(F)** Histological section under polarized light. The collagen fibers of the lamellar bone (LB) was oriented in a parallel way in many fields, and close to the biomaterial particles (P) (Acid fuchsin-Toluidine blue 40X).

After microCT testing the biopsy was available for histological evaluation. At low magnification, the sample revealed a complete integration of the scaffold, and only in the most peripheral portion, a small amount of soft tissue was present. Indeed, the residual biomaterial block, constituted by interconnected pores, was filled with bone. This portion was close to a thin layer of cortical bone with very small marrow spaces (at the bottom of the sample) (Figure 2A). At high magnification, the biomaterial was well-incorporated in the mature bone both in areas close to the cortical bone and in the areas far from it. At the bone-biomaterial particles interface, the particles showed a lower density compared to their central portion (Figure 2B). No gaps were detected at the bone-particles interface, and the bone was always in intimate contact with the particles. The porous structure of the biomaterial was partially modified, and the shape of the particles revealed signs of degradation. Moreover, in one field, close to the residual biomaterial, a multinucleated giant cell was observed, showing that the process of biomaterial resorption happened slowly over time (Figure 2C). In the small marrow spaces, some blood vessels were present, and in a few fields, foci of bone remodeling were observed (Figure 2D) with osteoblastic activity. No inflammatory cells were present. The percentage of bone, residual biomaterials and soft tissues was respectively 59.2, 25.6, and 15.2%.

### Polarized Light Observations

The same fields of the samples were examined under polarized light and compared to the light microscopic images in order to study the quality of the bone and the orientation of collagen fibers. In all cases, the biomaterial block was surrounded by lamellar bone with oriented parallel collagen fibers (Figures 2E,F), and only in small areas they were randomly oriented.

## Discussion

The purpose of the present study was to assess the healing and resorption process of BCP 3D printed bone substitute and the nature and amount of regenerated bone. The newly formed tissues were evaluated by an innovative experimental approach based on histological and X-ray high-resolution tomography (microCT) analysis. MicroCT was widely shown to be a powerful tool for scaffold characterization (Landi et al., 2000; John and Wenz, 2004; Iezzi et al., 2012, 2020; Giuliani et al., 2018a,b,c), obtaining not only a 3D image of a scaffold, but also providing qualitative and quantitative information on its structure (Renghini et al., 2013; Giuliani et al., 2014, 2016).

It is possible, starting from CBCT files, to create a 3D prototype of the patient's maxilla/mandible, obtained by transferring the files to specific reconstruction software (Mangano et al., 2015b; Luongo et al., 2016). Potent CAD software can design a custom-made bone graft straightforwardly on this 3D model (Figliuzzi et al., 2013; Mangano et al., 2015b; Luongo et al., 2016; Chung et al., 2020). The file of the 3D designed scaffold is sent to a computer-numeric-control (CNC) machine, which mills the custom-made bone graft of the chosen material (Figliuzzi et al., 2013; Mangano et al., 2015b; Luongo et al., 2016). Finally, the surgeon can easily adapt the customized scaffold in the surgical site, performing a straightforward and less time consuming surgery procedure, with reduced discomfort for the patient (Figliuzzi et al., 2013; Mangano et al., 2015b; Luongo et al., 2016; Kim et al., 2020). Micro- and macro-porous biphasic calcium phosphate (BCP) have been mainly recommended and characterized in oral surgery practices (Piattelli et al., 1996b; Mangano et al., 2013a, 2015b, 2019; Giuliani et al., 2014, 2016; Kim et al., 2020). They are produced by combining HA and beta-TCP in various compositions rates (HA/beta-TCP ratios), and represent the most important BCP ceramics for dental and medical applications (Piattelli et al., 1996b; Mangano et al., 2013a, 2015b, 2019). In literature, successful bone regeneration using biphasic calcium phosphate materials, both granules and blocks, has been reported in some clinical applications for maxillary sinus elevation (Mangano et al., 2013a; Giuliani et al., 2014; Ohayon, 2014). However, most of these reported studies are based on a single time point (6 months), not allowing an accurate assessment of the kinetics of bone growth on the long-term and thus inhibiting a detailed comparison between different morphologies of the scaffold (Scarano et al., 2000; Giuliani et al., 2016). Moreover, most of the existing studies report on 60% HA and 40% TCP, which is characterized by two types of porosity: macroporosity (pores with diameters range 300–600 micron) leads the colonization of ceramic by osteogenic cells, and microporosity (pores with diameters <10 micron) permits biological fluids circulation (Iezzi et al., 2012; Mangano et al., 2019). TCP dissolution leads to more space available for new bone formation, while the HA maintains its role as a scaffold (Mangano et al., 2015b). 3DP offers several advantages over other SFF techniques for scaffold production:

1. 3DP can make scaffolds with high consistency and precise structural anisotropy.
2. 3DP does not implicate high temperature, strong chemicals, and support structures.
3. The high constructing speed of the print head makes the mass production of scaffolds feasible.
4. It is possible to include biological mediators into the scaffolds if the binder is water.

Besides its chemical structure, one of the key parameters in 3D scaffolds is its internal configuration. Pore size would be directly associated to bone formation, since it offers surface for cell adhesion and space for bone ingrowth; pore interconnection would provide the way for cell distribution/migration and permit an efficient *in vivo* blood vessel development, suitable for bone tissue neo-formation and remodeling. Studies on non-human primates have shown bone formation by bioactive biomimetic matrices scaffolds (Ripamonti et al., 2008). Geometry is a series of recurring concavities that biomimetizes the remodeling cycle of the primate osteonic bone.

Recent studies have shown that microCT is a powerful tool for studying not only the microarchitecture of the jaw (Mangano et al., 2013b; Giuliani et al., 2016, 2018b), but also its osteocyte lacunar morphology and density (Giuliani et al., 2018b,c; Iezzi et al., 2020). In this context, it was observed in the BCP-based Test-sample an augmented specific volume and trabecular thickness, together with decreased specific surface and trabecular spacing, with respect to the unloaded control, and the peri-dental sites.

## Conclusions

Within the limitations of this study, based on only one patient, results indicate that while the usual unloaded jaw sites, the BCP-based Test-sample preserved a correct microarchitecture even after 7 years without masticatory loading. However, our investigation of the lacunar mean data indicated that the fact that the present specimen was unloaded for 7 years did not affect the mean volume and size of the osteocyte lacunae, but a lower lacunar density was found with respect to the peri-dental biopsies, confirming previous data (Iezzi et al., 2020).

More studies on human, with higher number of patients and with long follow up should be conducted to confirm data presented in the present paper.

## Data Availability Statement

The raw data supporting the conclusions of this article will be made available by the authors, without undue reservation.

## Ethics Statement

The studies involving human participants were reviewed and approved by The Ethical Committee of the Hospital of Varese, Italy approved the study protocol (N°826 del 03/10/2013). The patients/participants provided their written informed consent to participate in this study. Written informed consent was obtained from the individual(s) for the publication of any potentially identifiable images or data included in this article.

## Author Contributions

AP and CM: conceptualization. AP, CM, and GI: methodology. AG and MR: software. AG and GI: formal analysis. AG, MR, and GI: investigation and data curation. CM, AG, IDT, and GI: writing—original draft preparation. AP: writing—review and editing. AP and IDT: supervision. All authors have read and agreed to the published version of the manuscript.

## Conflict of Interest

The authors declare that the research was conducted in the absence of any commercial or financial relationships that could be construed as a potential conflict of interest. The reviewer AS declared a shared affiliation, with no collaboration, with the authors, IDT, AP and GI, to the handling editor at the time of the review.
